# Safety and preliminary efficacy of an electrically stimulated implant for mandibular bone regeneration: a pilot study in a large animal model

**DOI:** 10.1007/s00784-025-06303-7

**Published:** 2025-04-07

**Authors:** Peer W. Kämmerer, Nadja Engel, Rainer Bader, Vivien Engel, Bernhard Frerich, Diana Heimes, Justin Kröger, Laura Lembcke, Franz Plocksties, Hendrikje Raben, Ursula van Rienen, Armin Springer, Dirk Timmermann, Julius Zimmermann, Michael Dau

**Affiliations:** 1 Department of Oral and Maxillofacial Surgery, Facial Plastic Surgery, University Medical Center, Johannes Gutenberg University Mainz, Augustusplatz 2, 55131 Mainz, Germany; 2 Department of Oral and Maxillofacial Surgery, Facial Plastic Surgery, Rostock University Medical Center, Schillingallee 35, 18057 Rostock, Germany; 3 Oscar Langendorff Institute of Physiology, Rostock University Medical Center, Gertrudenstrasse 9, 18057 Rostock, Germany; 4 Research Laboratory for Biomechanics and Implant Technology, Department of Orthopaedics, Rostock University Medical Center, Doberaner Str. 142, 18057 Rostock, Germany; 5 Institute of Chemistry, Universität Rostock, Albert-Einstein-Strasse 3a, 18059 Rostock, Germany; 6 Institute of Applied Microelectronics and Computer Engineering, University of Rostock, Albert- Einstein-Str. 26, 18119 Rostock, Germany; 7 Institute of General Electrical Engineering, University of Rostock, Albert-Einstein-Straße 2, 18059 Rostock, Germany; 8 Medical Biology and Electron Microscopy Centre, University Medical Center Rostock, Strempelstraße 14, 18057 Rostock, Germany

**Keywords:** Mandibular bone defect regeneration, Electrical stimulation in bone healing, Large animal model, Critical-size defect healing, Bioelectrical stimulation, Preclinical evaluation of implant-based bone regeneration

## Abstract

**Objectives:**

Large mandibular defects present challenges for bone regeneration. This pilot study evaluates the safety and preliminary efficacy of direct electrical stimulation (ES) on tissue healing in a preclinical model, testing whether ES can enhance bone formation in critical-size mandibular defects.

**Materials and methods:**

Six adult mini pigs with critical-size mandibular defects were used in a split-mouth design. The test group (*n* = 6) received 0.5 V AC/20 Hz ES for 3 × 45 min daily over three weeks, while the control group (*n* = 6) had no stimulation. Safety, early bone growth, and soft tissue effects were assessed at three locations: S1 (cancellous bone interface), S2 (middle of the defect), and S3 (pristine dense bone).

**Results:**

The ES group showed no adverse effects, confirming implant safety. The ES group exhibited significantly higher bone formation, particularly in S2 and S3. Enhanced vascularization and immune response, in terms of increased mast cells, were also observed in S2.

**Conclusions:**

The implant device with ES is safe and promotes bone formation and vascularization in select sub-regions (S2 and S3). However, ES alone may not suffice for complete bone regeneration in critical-sized defects, and further optimization is needed.

**Clinical relevance:**

This study demonstrates the potential of ES to improve bone healing in large mandibular defects, offering insights for clinical use in maxillofacial reconstruction.

**Supplementary Information:**

The online version contains supplementary material available at 10.1007/s00784-025-06303-7.

## Introduction

Treating critical-size mandibular bone defects arising from trauma or tumor diseases poses a substantial therapeutic hurdle. Despite considerable research, autologous bone transplants remain the preferred approach due to their unparalleled osteoinductive properties, which current alloplastic, allogeneic, or xenogeneic materials have yet to replicate [1, 2]. Nonetheless, the inherent limitations in the physiological availability of autologous bone and the necessity for a secondary surgical intervention to harvest bone from the donor site impose significant burdens on patients [2].

Bone tissue engineering aims to replicate the intricate bone microenvironment, encompassing its biochemical and biophysical stimulating factors [3, 4]. Since natural bone displays piezoelectric properties, using smart materials holds promise in generating electric microenvironments within the bone, thereby triggering biological cellular responses through mechanotransduction [5]. Notably, endogenous electrical fields play pivotal roles in governing various biological tissues’ development, maintenance, repair, and regeneration [4, 6].

Electrical stimulation (ES) has emerged as a promising modality in promoting bone healing processes, encompassing fracture healing, bone remodeling, and applications in orthopedics, maxillofacial surgery, and dental implantology [7–14]. The efficacy of ES primarily lies in its capacity to elicit biochemical and physiological responses through the application of electric fields [15, 16]. In direct ES, wherein electric fields are generated via applied voltage, conductive electrodes are strategically positioned near the target area to deliver electrical current directly to the tissue by establishing a voltage gradient between the anode and cathode. However, this method may inadvertently produce undesirable by-products, such as hydrogen peroxide and free radicals, while posing challenges related to unpredictable fluctuations in current density stemming from variations in tissue impedance during stimulation [17]. Despite its potential, the precise mechanisms underlying electrically induced osteogenesis require further elucidation [18], constraining ES’s widespread clinical adoption for bone regeneration.

Our research group has previously characterized and extensively tested the ES device employed in this study through numerical simulations [19–21]. It has demonstrated efficacy across various cell types, including chondrocytes, mesenchymal stem cells [22–25], bacteria [23], and osteoblasts [23, 26–28]. Primarily utilizing the ASNIS III s-series screw system (Stryker GmbH, Duisburg, Germany), which is clinically applied in the treatment of avascular femoral head necrosis [29], our previous experiments have shown effective alternating current (AC) stimulation of osseous cells using 20 Hz sine waves with fixed root mean square values ranging from 0.2 to 1.7 V [23–25]. Conversely, higher electrical stimulation with 2.8 V did not exhibit favorable in vitro effects [30].

The subsequent phase in transitioning bone ES for clinical applications involves preclinical characterization through adequate in vivo models. Despite the favorable outcomes of ES in bone-related applications, research into its utilization in craniofacial critical-size defects, particularly within large animal models, is notably lacking. Thus, this study aims to conduct a pilot study to assess the safety of integrating an implant system capable of endogenous ES into a critical-size mandibular defect. Additionally, this study will evaluate the preliminary efficacy of ES on bone formation compared to non-stimulated controls. The null hypothesis posits no significant difference in bone healing between ES and non-ES-treated mandibular critical-size defects.

## Materials and methods

### Electrically active implant

**Fig. 1:**
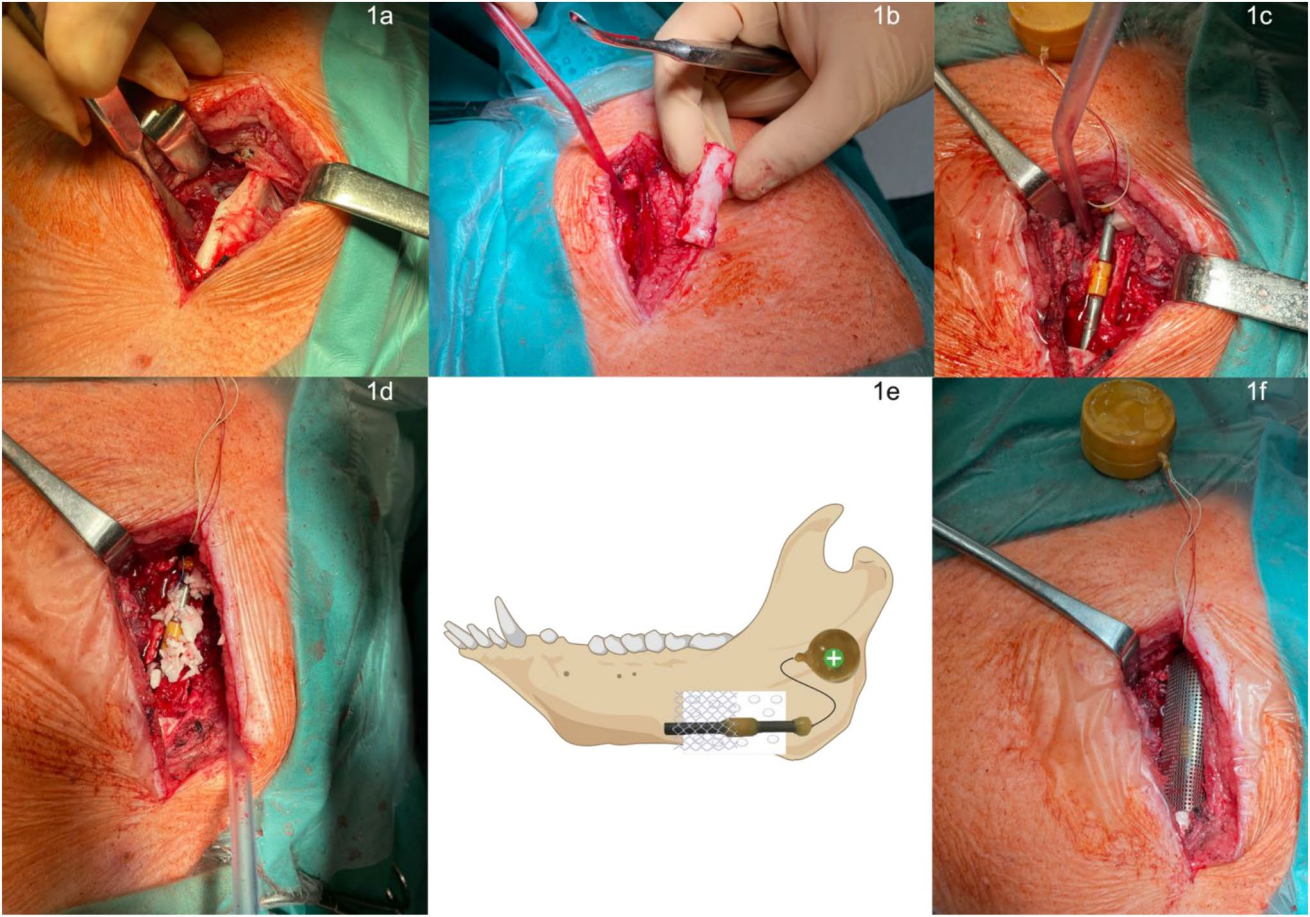
Surgical workflow illustrating the experimental procedure. (**a**) Exposure of the mandibular bone via a submandibular approach. (**b**) Creation of a standardized critical-size defect by bone removal. (**c**) Insertion of the ES implant and stimulation device within the defect. (**d**) Placement of the ES implant within the defect, including the ground bone. (**e**) Schematic representation of the inserted ES implant, battery, bone particles, and titanium mesh. The mesh fully covers the defect, but the image is designed to show the presence of bone particles within the defect beneath the mesh. (**f**) Final positioning of the ES implant with the titanium mesh securing the defect

The electrically active implant (ES) used in this study was previously validated through a combination of numerical simulations, in vitro experiments, and preclinical assessments. Numerical modeling studies optimized the electrode configuration and electrical field distribution to ensure effective stimulation while maintaining biocompatibility [19, 20]. Further, in vitro studies demonstrated that alternating current (AC) stimulation in the applied range enhances osteoblast adhesion, proliferation, and differentiation [25, 27]. Additionally, corrosion resistance and encapsulation stability were tested to confirm the long-term functionality of the implant in biological environments [31, 32]. These prior validations established the feasibility and safety of the implant, allowing its application in the present large animal study. The ES device has an approximate length of 7,5 cm and a diameter of 1.8 mm. It comprised a biocompatible core structure with conductive components to enable electrical stimulation. In brief, it consists of two titanium (cp. Ti grade 4) components separated by an isolator crafted from polyether ether ketone (PEEK; Fig. 1c & e). Several steps were undertaken before its application in animal experiments to ensure the device’s functionality and biocompatibility. The implant’s wires, circuit board [32], and battery underwent encapsulation [31] to enhance corrosion resistance for a minimum of 100 days. The primary objective was to ensure the device’s corrosion resistance for at least 100 days. The wires were encased in polyvinyl chloride (PVC; Soprema GmbH, Mannheim, Germany), while the battery and circuit board were shielded with Polytec EP 655 (Polytec PT, Karlsbad, Germany). Additionally, to further enhance biocompatibility, a comprehensive coating of RTV silicone MED-1511 (NuSil, Carpinteria, CA, USA) was applied. Before in vivo implantation, the device underwent sterilization through gas plasma treatment involving 210 g H2O2 and a high-frequency field (100–1200 W; Steriplas 2000, Heinz Meise GmbH, Schalksmühle, Germany).

The primary outcome of this study was to assess the safety of the electrically stimulated implant, including the absence of severe inflammatory reactions, implant-associated complications, or adverse tissue responses. Secondary outcomes included bone formation, vascularization, and inflammatory response, which were evaluated through histological, histomorphometric, and immunohistochemical analyses.

### Animal experiments

Approval for the animal experiment was obtained from the State Office for Agriculture, Food Safety and Fisheries Mecklenburg-Vorpommern, Germany (file number: 7221.3-1-035/19). All animal procedures were conducted at the Core Facility Central Animal Facility, Rostock, Germany, in compliance with relevant regulations and guidelines. The animal experiments adhered to the ARRIVE guidelines and followed the U.K. Animals (Scientific Procedures) Act, 1986, associated guidelines, and EU Directive 2010/63/EU for animal experiments (Supplement 1).

A fourteen-day adaptation period was allotted for the animals to acclimate to the new husbandry conditions, staff handling, and dietary regimen before the experiments, ensuring minimized stress and promoting baseline stability. The quantity of feed provided was adjusted to meet the animals’ maintenance requirements and was based on their respective body weight. Water was available ad libitum, and the animals were maintained under a defined day/night rhythm.

For the analyses, six female adult (at least two years of age) Aachener minipigs (European Union Trademark #017715434; Gerd Heinrichs, Heinsberg, Germany) with a total of 12 implants (electrostimulation (test; *n* = 6), and no electrostimulation (control; *n* = 6)) were used in a randomized split-mandible model using www.random.org.

The intramuscular premedication was carried out in the following order and dosage:

1. Azaperone (Stresnil^®^, Richter Pharma AG, Weis, Austria; 7–8 mg/kg body weight) together with ketamine hydrochloride 10% (Ketaset^®^, Zoetis, Parsippany, New Jersey, USA; 17.5–20 mg/kg body weight), and.
2. Ketamine hydrochloride 10% (33.5 mg/kg body weight) and midazolam (Richmond Vet Pharma, Singapore; 0.19 mg/kg body weight).

In the operating room, the animals received an i.v. access, and by putting on the ventilation hose, they were saturated with 40% oxygen for at least 2 min (O_2_ saturation > 98%). After the endotracheal intubation, the ventilation parameters were set with the target value of an end-tidal CO_2_ of 3.5–4.5 kPa and a respiratory rate of 13–20 times per minute. Immediately after induction of anesthesia, the animals were given i.v. carprofen (Rimadyl^®^, Zoetis; 4 mg/kg body weight).

The bilateral access area at the jaw angle of the lower jaw was shaved, disinfected, and sterilely covered. The cut areas were marked with a skin marker. Lidocaine with epinephrine 0.001% (Xylocitin^®^, mibe GmbH Arzneimittel, Sandersdorf-Brehna, Germany; 10 ml 2%) was used for local anesthesia. After a submandibular incision, the mandibular bone was exposed directly before the insertion of the M. masseter (Fig. 1a). Following the demarcation of the intended bone defect (1.5 × 3.5 × 1.5 cm), the defect was surgically created using a round bur cooled with NaCl; the bone was removed and ground into particles (Fig. 1b). The implant was placed centrally within the defect, maintaining an average distance of approximately 1–2 mm from the surrounding bone. This positioning ensured uniform exposure to the defect environment while preventing direct mechanical contact with the bone walls (Fig. 1c). A pocket was carefully prepared along the medial border of the mandible beneath the masseteric muscle, where the stimulation device was positioned later on. The defect holding the implant was filled with the ground bone (1d) and encased with a titanium mesh (Henry Schein, Melville, New York, USA, Fig. 1e & f). Subsequently, a multi-layer wound closure procedure was performed using both absorbable (Vicryl^®^ 3 − 0, Ethicon, Norderstedt, Germany) and non-absorbable suture materials (Resolon^®^ 3 − 0, Resorba Medical GmbH, Nürnberg, Germany). The wound was then coated with silver spray (Agrochemica GmbH, Bremen, Germany). After the intervention, postoperative X-ray assessments were carried out, and the animals were transferred to individual stalls for observation. A fully functional ES implant was placed and activated in the test group. In the control group, an identical implant was inserted but without electrical activation, ensuring that any observed effects could be attributed to electrical stimulation rather than the mere presence of the implant. The placement and fixation procedures were identical in both groups.

### Electrostimulation

Electrostimulation, administered via sinusoidal alternating electric fields with an amplitude of 0.5 V (peak value) and a frequency of 20 Hz, was conducted in three sessions lasting 45 min each per day. The stimulation commenced 48 h after surgery. The functionality of the stimulation unit was monitored without direct contact, eliminating the need for sedation of the animals, as previously described [32]. The electrical stimulation parameters used in this study (0.5 V AC, 20 Hz) were numerically optimized and validated in previous studies, ensuring a biologically favorable electric field strength for promoting bone healing [20, 21, 25]. They were selected based on prior in vitro and in vivo research demonstrating their effectiveness in promoting osteogenesis while minimizing adverse effects. Alternating current (AC) stimulation in the 0.2–1.7 V range at 20 Hz has enhanced osteoblast activity, mesenchymal stem cell differentiation, and early bone formation in preclinical models. In contrast, higher voltages (> 2.8 V) have been associated with increased oxidative stress and potentially adverse effects on bone regeneration [23–25, 30].

### Postoperative care

In cases of uneventful wound healing, group housing was reinstated starting the first week after the surgical procedure. During the initial seven postoperative days, 40 drops of metamizole (Novaminsulfone, Dechra Veterinary Products GmbH, Aulendorf, Germany; 500 mg/ml) were administered via the feed in the morning and evening. If signs of pain were observed (e.g., restricted movement, reduced food intake), pain medication was administered individually via intramuscular injection of 4 mg carprofen per kg body weight every 24 h. The residual amounts of distributed food were measured to detect any pain-related reduction in food intake by the animals. A 3-week healing period was chosen to evaluate the early-phase tissue response, including wound healing, inflammatory reaction, and initial bone remodeling. This timeframe is well established in preclinical models for assessing the biocompatibility of novel implant materials. Given that key markers of implant safety, such as inflammatory cell infiltration and early osteogenesis, can be reliably assessed within this period, this study focused on short-term tissue response rather than long-term bone regeneration. After the respective healing period of three weeks under electric stimulation, the animals were euthanized under deep sedation using a mixture of azaperone, ketamine hydrochloride, and midazolam, followed by a final overdose of pentobarbital (Euthasol^®^, Virbac, Carros, France). For this purpose, all animals received 450 mg/10 kg body weight.

### Sample preparation

**Fig. 2:**
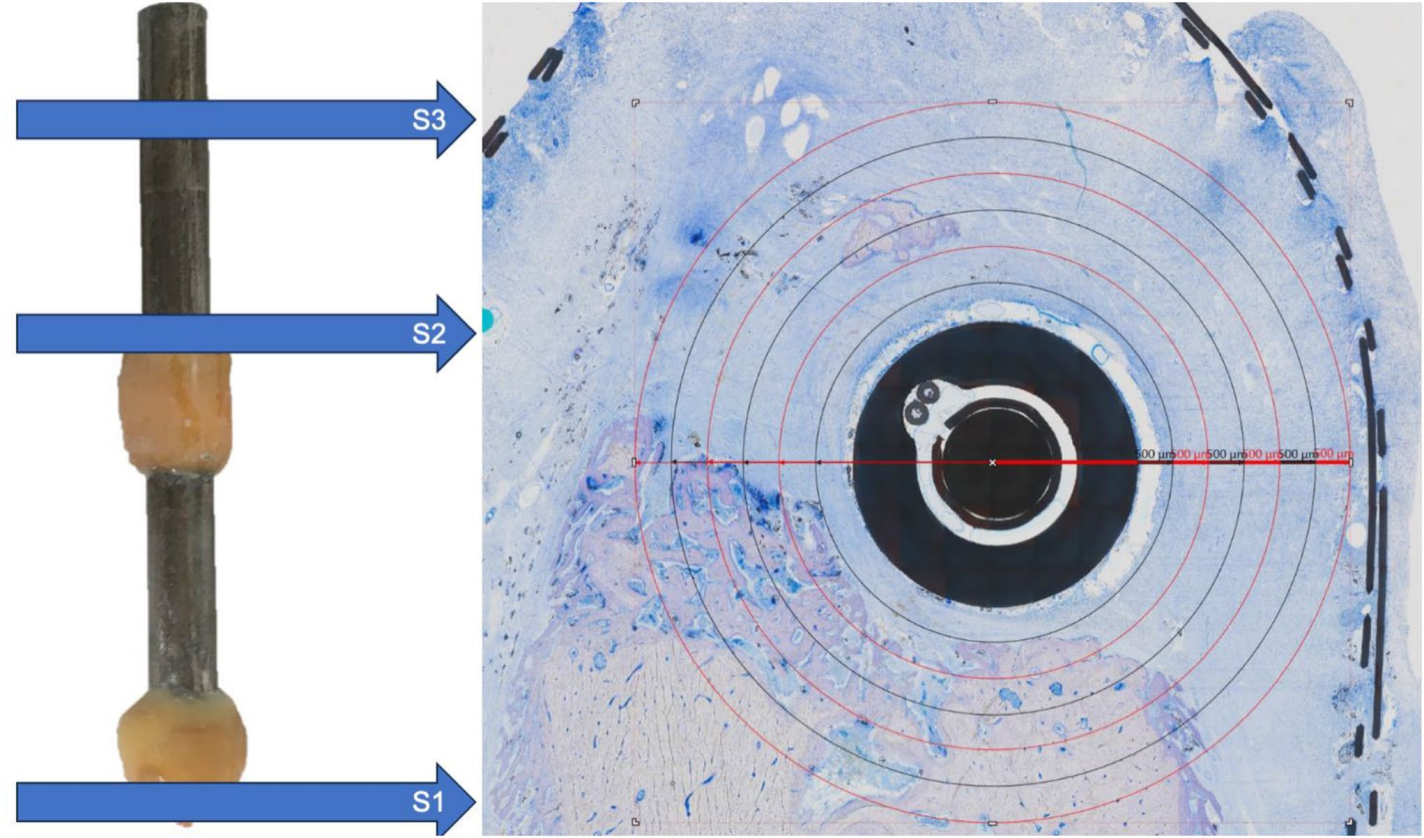
(**a**) Each implant was examined in three specific regions labeled S1, S2, and S3. S1 and S3 are situated towards the posterior and anterior parts of the mandible, respectively. At all sites, new bone formation around the implant (here: ES implant at S2) was analyzed in sections transverse to the implant (Toluidine Blue and Pyronine Yellow; original magnification x10). A total region of interest (ROI) extending 3 mm from the implant was analyzed using ImageJ. For subgroup analysis, the extent of newly formed bone within circular regions surrounding the implant, each with a radius of 500 μm (indicated by red and black circles), was measured and compared across the respective experimental groups

The preparation of samples followed the established protocol as previously outlined [33, 34]. In brief, specimens were obtained from the relevant bone segments using an Exakt 310CP diamond band saw (Diamond Cutting Band Type 310, 0.1 mm D64; Exakt, Hamburg, Germany) and ground to a thickness of 5 mm, transverse to the implants’ stimulation unit. The bone slices were immediately embedded in PMMA (Technovit 7100, Heraeus Kulzer, Hanau, Germany). Grinding to a 30 to 50 μm thickness was completed using an Exakt 400CS micro grinding device with 1200 grit sandpaper for initial reduction and 4000 grit for polishing. This process yielded bone slices situated at three distinct locations: at the interface between residual bone and the defect within the cancellous bone (S1), within the middle of the defect (S2), and the pristine bone with surrounding bone tissue (S3; Fig. 2). The obtained slices were promptly embedded in polymethyl methacrylate (Technovit^®^ 7200; Heraeus Kulzer, Hanau, Germany) and subsequently cut to a thickness ranging from 30 to 50 μm. All specimens were subjected to Toluidine Blue and Pyronine Yellow staining (Morphisto Ltd., Offenbach am Main, Germany) [35] and analyzed using a DM8000 M microscope (Leica Microsystems, Heidelberg, Germany). Furthermore, the slices were subjected to analysis using a field emission scanning electron microscope (FE-SEM, MERLIN^®^ VP Compact) equipped with an energy dispersive X-ray (EDX) detector (XFlash 6/30) and Quantax Esprit 2.0 analysis software (Co. Bruker, Berlin, Germany). The elemental distribution within representative areas of the slices was mapped based on the EDX-spectra data using the Quantax Esprit Microanalysis software (version 2.0).

### Safety assessment

The safety of the ES implant was a primary consideration in this study. Throughout the experimental period, the animals were monitored for any signs of adverse reactions or complications related to the implants. This included regular veterinary check-ups to assess overall health and wound healing. Postoperative X-rays were conducted to evaluate the positioning and integrity of the implants.

### Evaluation

After three weeks, a tissue response to the implants was intended to be evaluated in a cohort of six animals with six implants for each group. Any postoperative hematomas were expected to be resolved at this juncture, enabling a histological examination of the tissue surrounding the implant. In detail, this assessment should allow for examining the early peri-implant bone formation induced by the procedure. Personnel involved in outcome assessment and data analysis were blinded to group assignments to mitigate potential bias.

Early peri-implant bone formation was assessed at several locations through histological methods [34, 35]. To accurately assess new bone growth, it was essential to distinguish between the “old” cortical bone inserted into the defects at the time of surgery and the newly formed woven bone. Old cortical bone, harvested and inserted into the defects during surgery, exhibited characteristic features of mature bone, including organized lamellar structure and minimal cellular activity. In contrast, new woven bone displayed a more irregular and disorganized matrix, indicative of early bone formation. A higher density of osteocytes and a prominent vascular network, typical features of woven bone during the initial stages of bone healing, characterized the newly formed bone. Toluidine Blue and Pyronine Yellow staining was employed to enhance the visualization of these differences. Old cortical bone stained more intensely and uniformly due to its dense, calcified nature, while new woven bone appeared lighter and more diffuse, reflecting its immature and rapidly remodeling state. A total region of interest (ROI) extending 3 mm from the implant was analyzed using ImageJ (Fig. 2). For subgroup analysis, the extent of newly formed bone within the circular regions surrounding the implant, each with a radius of 500 μm, was measured and compared across the respective experimental groups. New bone formation was quantified as the percentage of new bone content within each selected ROI. The total volume corresponded to the total area of the ROI. These values were obtained for each implant within three designated areas (S1-S3; Fig. 2). For supplemental descriptive analyses, additional soft tissue parameters (vascularization (amount of brownish stain in a distance up to 2 mm from the implant to total volume (%)), and inflammation (amount of mast cells in a distance up to 2 mm from the implant to total volume (%)) were assessed.

### Statistics

The ES implant’s novel design and innovative mechanism of action for mandibular bone defects presented challenges in estimating the expected differences between experimental and control groups. As no prior data were available, formal power calculations were not feasible. This study was therefore conducted as an exploratory pilot study, and the sample size was determined based on practical considerations and feasibility within the given experimental framework.

Due to the split-mouth study design, the statistical analysis was adjusted to account for the dependence between test and control sites. Continuous variables were first tested for normality using the Shapiro-Wilk test.

- Normally distributed data are presented as mean ± standard deviation (SD) and analyzed using the paired t-test.
- Non-normally distributed data are reported as median with interquartile range (Q1–Q3) and analyzed using the Wilcoxon signed-rank test.

All statistical analyses were conducted using Prism version 9.5.1 for Macintosh (GraphPad Software, Boston, MA, USA). The graphical representations clearly marked all significant differences (*p* ≤ 0.05) between test and control conditions. Scatter plots with individual data points, mean ± SD, and error bars were used to visualize the distribution of continuous data.

## Results

### Safety assessment

**Fig. 3:**
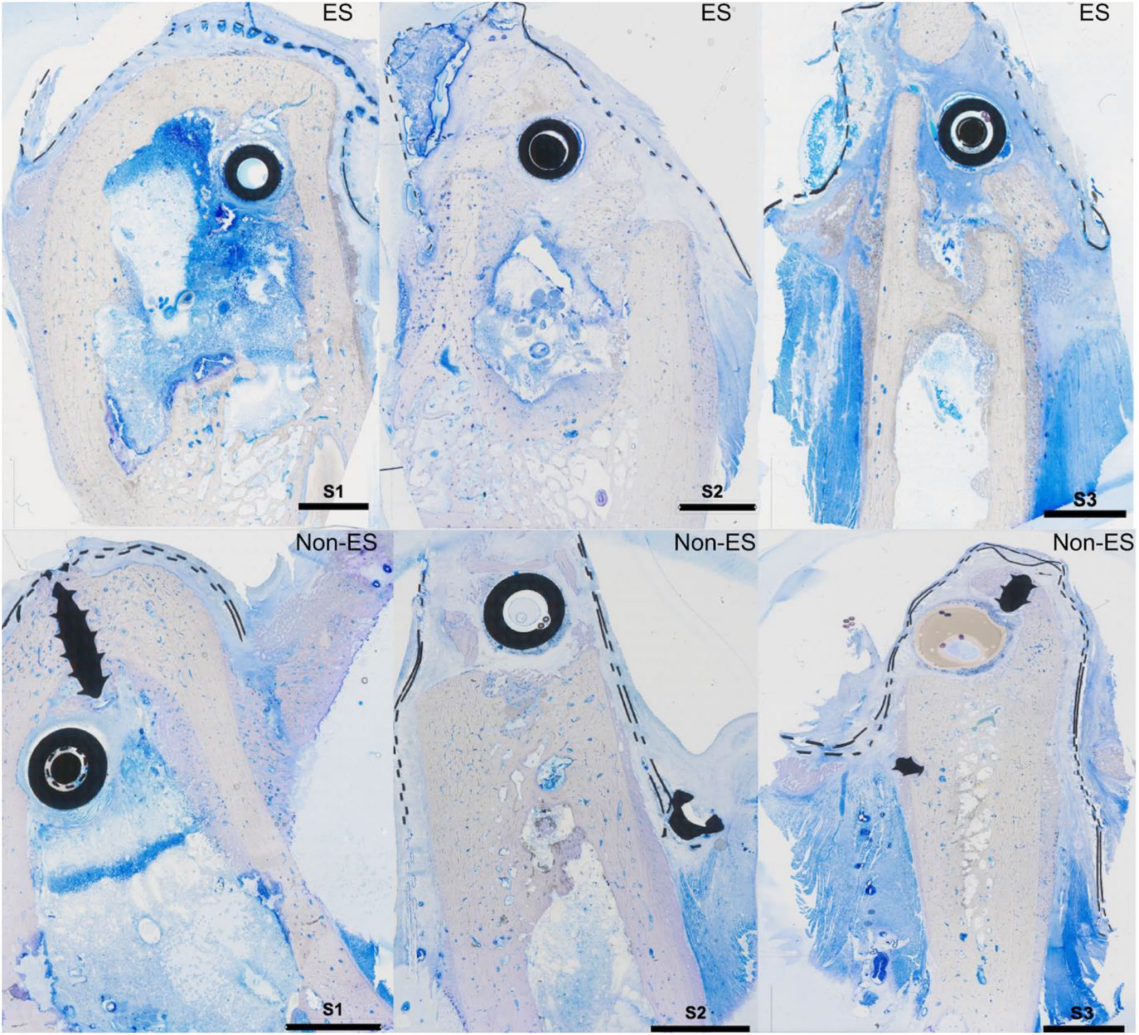
Representative histological slides (sections transverse to the implant; Toluidine Blue and Pyronine Yellow; original magnification x10) depicting an ES and non-ES implant three weeks after the start of ES at the locations S1, S2, and S3 from one animal. The corresponding locations of the analysis are given in Fig. 2, and the artificial material on top is the implant device

**Fig. 4:**
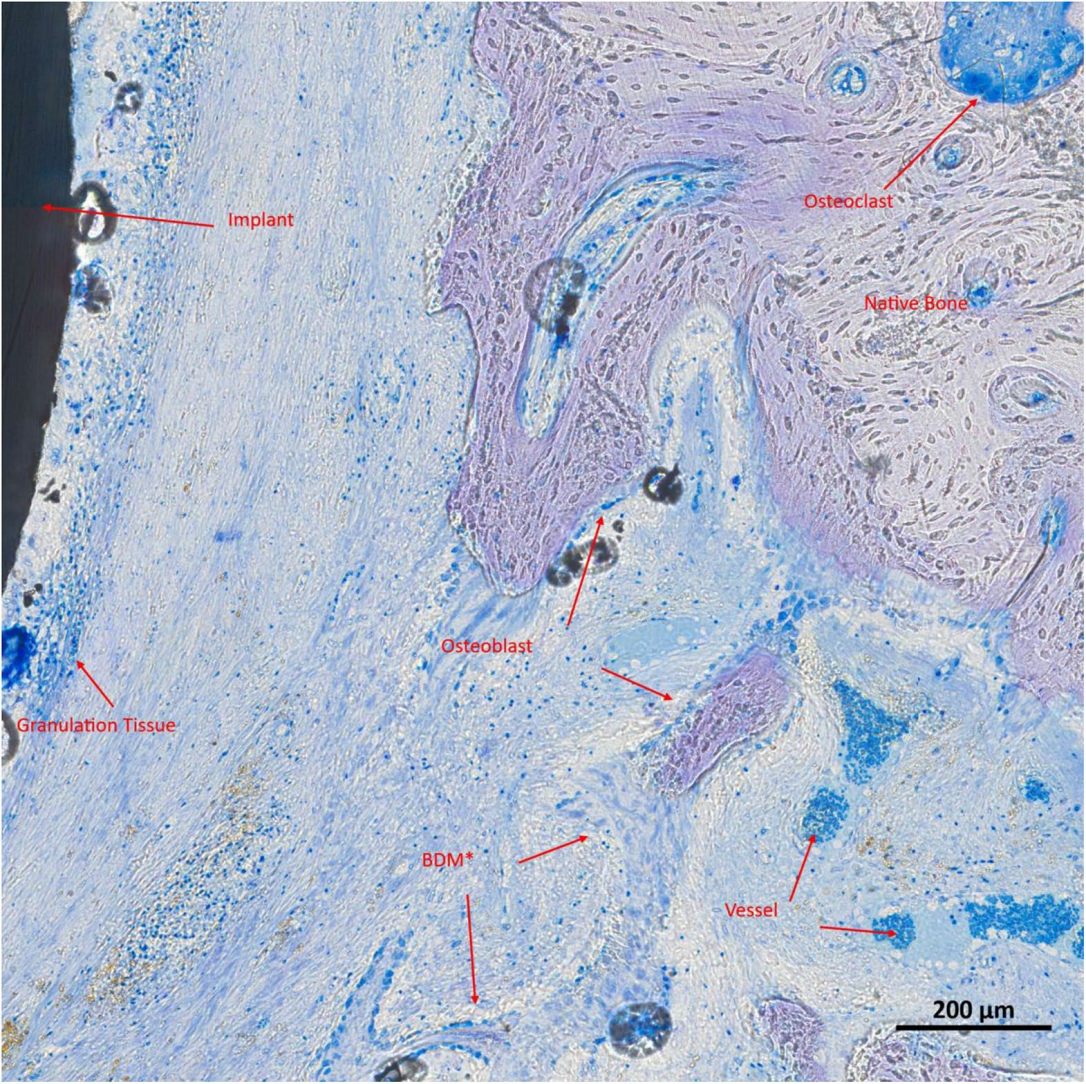
High-resolution image of a histological slide at S2 (sections transverse to the implant; Toluidine Blue and Pyronine Yellow) depicting an ES implant. For better understanding, the different structures are marked and described in red. In brief, maturing bone in the proximity of the implant is seen (BDM = bone in different phases of mineralization)

**Fig. 5:**
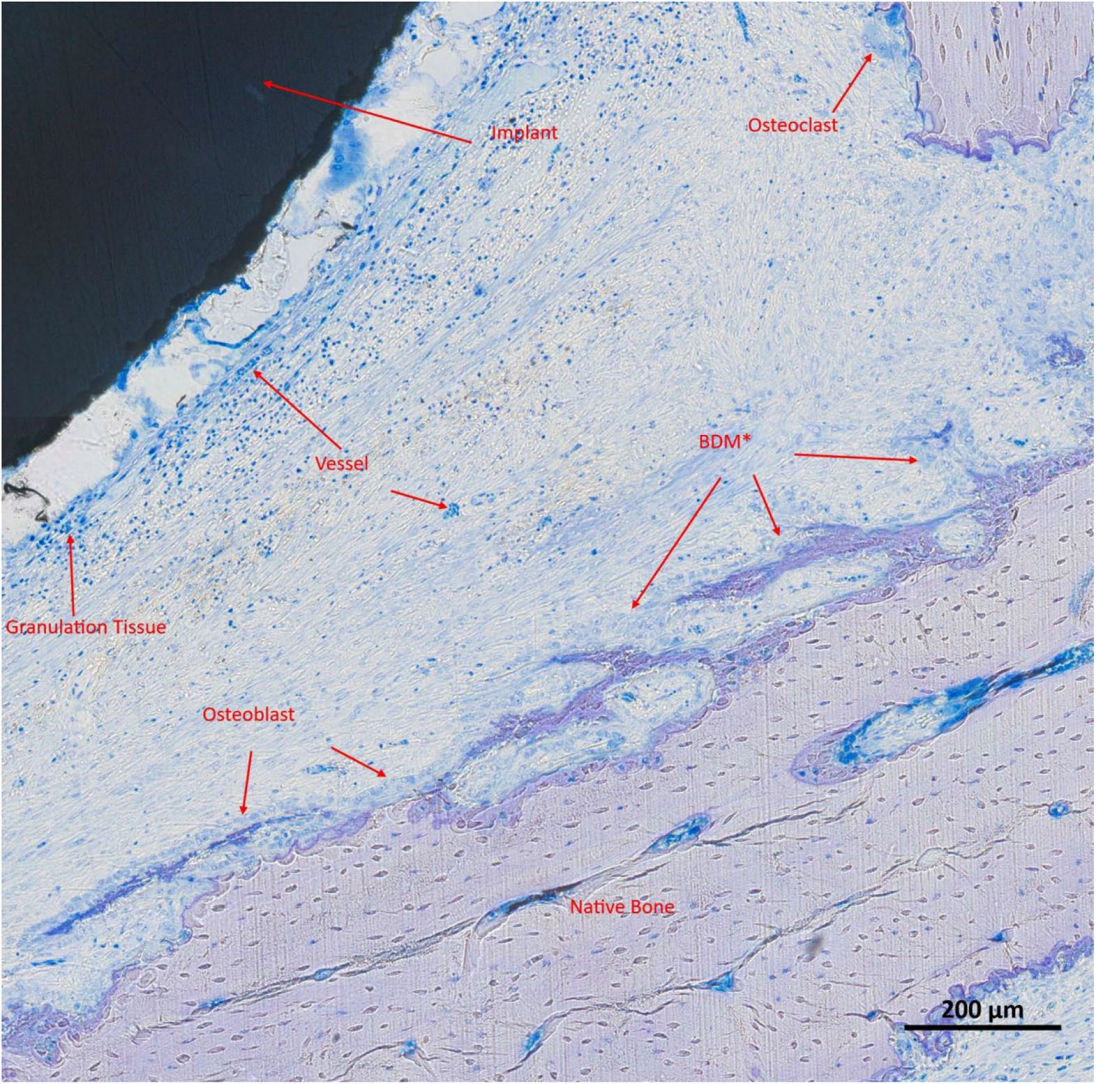
High-resolution image of a histological slide at S2 (sections transverse to the implant; Toluidine Blue and Pyronine Yellow) depicting a non-ES implant; visible structures are marked and described in red. In brief, less maturing bone in proximity of the implant is seen when compared to the ES implant (BDM = bone in different phases of mineralization)

**Fig. 6:**
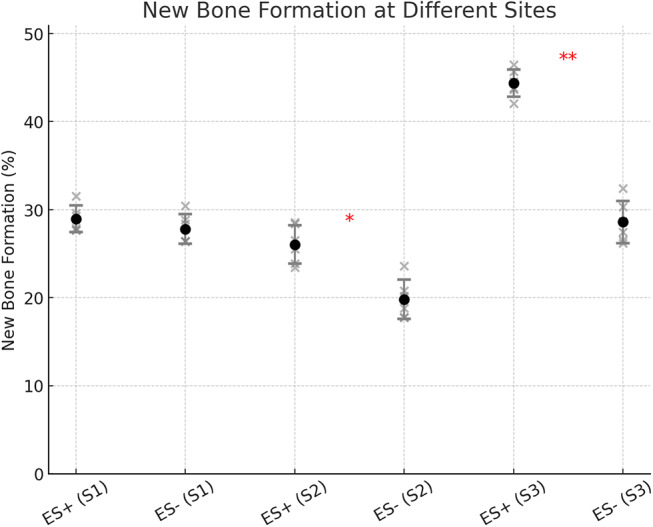
New bone formation at different sites (S1, S2, S3) in the ES+ (electrostimulated) and ES- (control) groups. The scatter plot displays individual values, with error bars representing each condition’s mean ± standard deviation (SD) of new bone formation (%). Significant differences between ES + and ES- groups are indicated with asterisks (*). Notably, significant differences were observed at S2 (*p* = 0.0097) and S3 (*p* < 0.00005), with ES + showing a higher mean bone formation compared to ES-. No significant differences were detected at S1 (*p* = 0.197)

In two of the six animals, wound-healing complications emerged three and four days after surgery, affecting one ES implant and one non-ES implant. Both cases required additional surgical exploration, during which a stimulation unit dislocation was identified in both cases and addressed by re-fixating it using the masseteric muscle. Following this intervention, no significant adverse effects were observed in the animals receiving the ES implants, indicating the device’s safety. Despite these challenges, all animals were included in the final histological assessment (Figs. 3, 4, 5 and 6). Importantly, no significant differences in bone formation or inflammatory response were observed between animals with and without initial wound-healing complications, indicating that these events did not introduce a measurable bias into the results.

### Descriptive and quantitative histological and histomorphometric analysis

Each defect was evaluated based on up to two histological slides, which together provided information on all three regions (S1, S2, S3). For descriptive analysis, a dense granulation tissue band approximately 2 to 3 layers thick was observed surrounding the electrode, transitioning into connective tissue containing residual infiltrates, including hematopoietic cells, identifiable by brown-stained erythrocytes. Bone formation predominantly appeared to originate from the pre-existing bone, with no signs of new bone formation adjacent to or arising from the implant. Vascularization remained intact, with no apparent disruptions in neovascularization. Compared to the non-ES implants, a tendency towards reduced osteoblast activity near the implant, but densification of the bone, was observed in the ES group. In the ES group, the native bone appeared less ragged at the contact surface with the newly formed bone. Also, in the ES group, larger intact vessels with hematopoietic cells were seen in the area of newly formed bone, but fewer small capillary vessels were present. Furthermore, in the stimulation group, the granulation tissue was not as strongly detached from the implant and was somewhat reduced (Figs. 4 and 5).

All data were normally distributed for quantitative analysis, as confirmed by the Shapiro-Wilk test. Consequently, statistical comparisons were conducted using paired t-tests, and results are presented as mean ± standard deviation (SD).

At S1, representing the interface between the residual cancellous bone and the defect, the ES group exhibited a mean new bone formation of 28.42% (SD: 3.89%), compared to 27.88% (SD: 3.64%) in the non-ES group (*p* = 0.197; Fig. 6). At S2, located in the middle of the defect, the ES group demonstrated a significantly higher new bone formation of 27.95% (SD: 4.12%), compared to 20.79% (SD: 4.32%) in the control group (*p* = 0.01; Fig. 6). At S3, within the pristine dense bone, the ES group exhibited a new bone formation of 45.62% (SD: 4.01%), whereas the non-ES group showed only 28.92% (SD: 3.94%) (*p* < 0.001; Fig. 6).

### Supplemental descriptive analyses

**Fig. 7:**
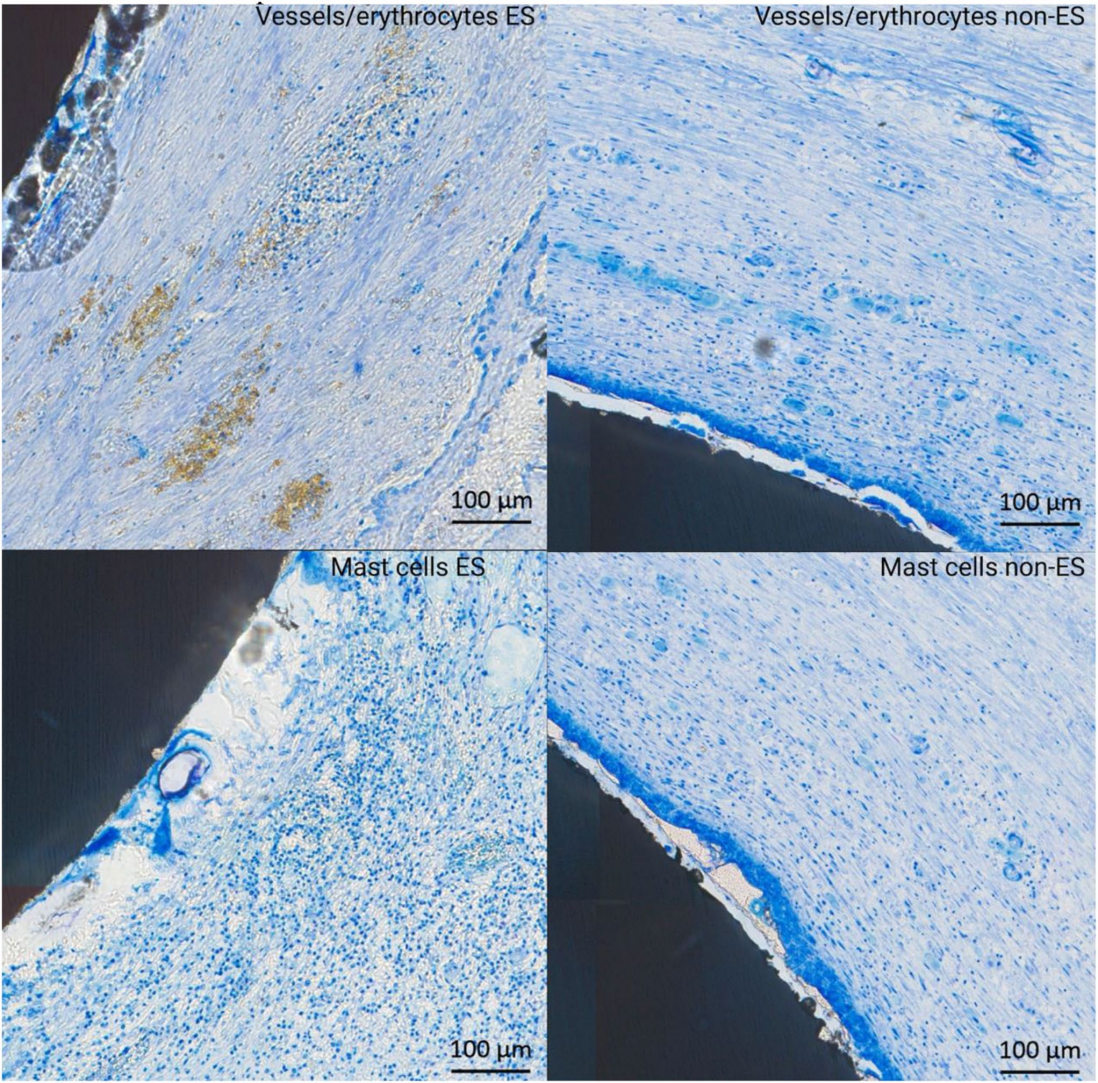
Representative histological slides (sections transverse to the implant; Toluidine Blue and Pyronine Yellow; original magnification x10) showing vessels/erythrocytes (**a**) and mast cells (**b**) around an ES and a non-ES implant three weeks after insertion at location S2

**Fig. 8:**
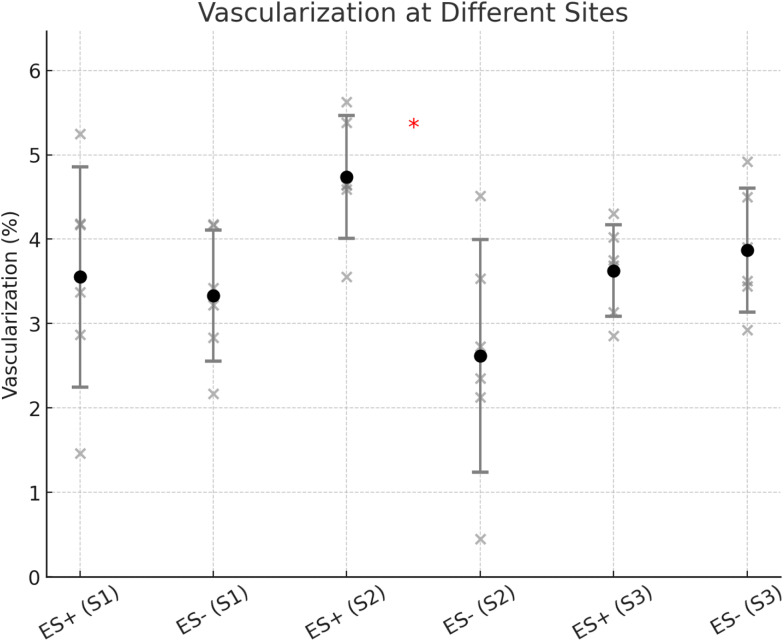
Vascularization at different sites (S1, S2, S3) in the ES+ (electrostimulated) and ES- (control) groups. The scatter plot displays individual values, with error bars representing each condition’s mean ± standard deviation (SD) of vascularization (%). Significant differences between ES + and ES- groups are indicated with asterisks (*). Notably, a significant difference was observed at S2 (*p* = 0.036), with ES + showing a higher mean vascularization than ES-. No significant differences were detected at S1 (*p* = 0.746) and S3 (*p* = 0.423)

For vascularization, all groups followed a normal distribution (*p* > 0.05 for all). In S2, a significantly higher number of vessels/erythrocytes was observed in the stimulated group compared to the controls (4.74% (SD: 0.73%) versus 2.62% (SD: 1.38%); *p* = 0.036; Figs. 7 and 8). However, in S1 (3.55% (SD: 1.31%) vs. 3.33% (SD: 0.78%); *p* = 0.746) and S3 (3.63% (SD: 0.54%) vs. 3.87% (SD: 0.74%); *p* = 0.423), no significant differences were found between the groups.

**Fig. 9:**
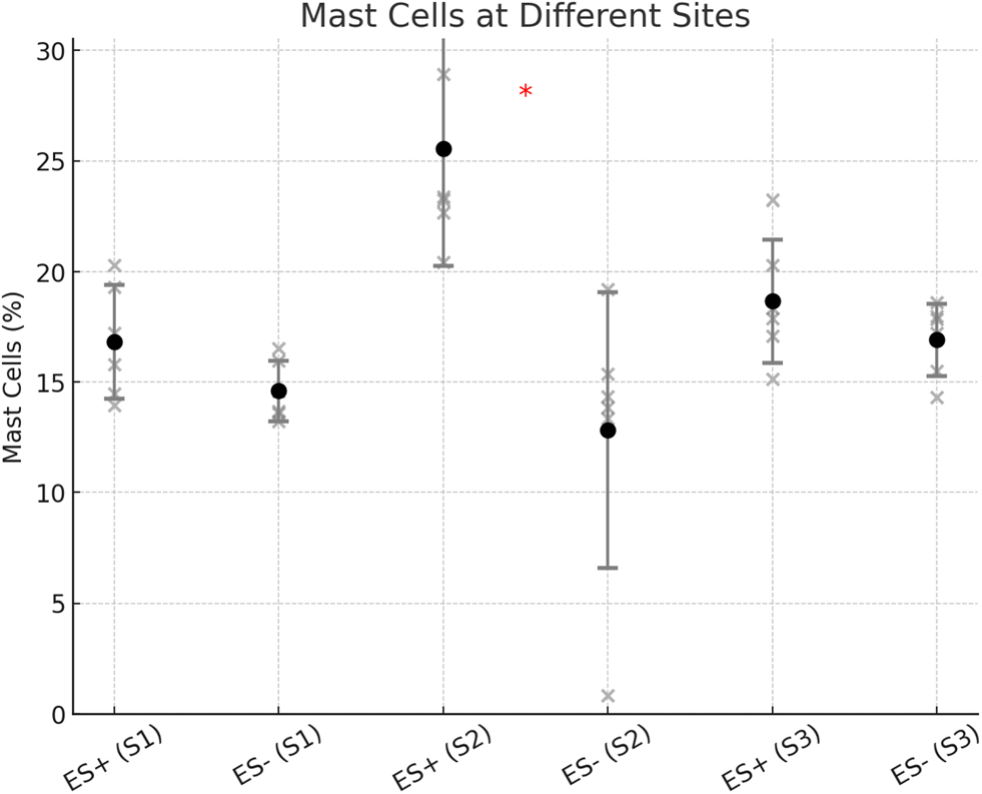
Mast cell presence at different sites (S1, S2, S3) in the ES+ (electrostimulated) and ES- (control) groups. The scatter plot displays individual values, with error bars representing each condition’s mean ± standard deviation (SD) of mast cells (%). Significant differences between ES + and ES- groups are indicated with asterisks (*). Notably, a significant difference was observed at S2 (*p* = 0.023), with ES + showing a higher mast cell count than ES-. No significant differences were detected at S1 (*p* = 0.188) and S3 (*p* = 0.141)

For mast cells, all groups followed a normal distribution (*p* > 0.05 for all). In S2, a significantly higher number of mast cells was observed in the stimulated group compared to the controls (25.54% (SD: 5.27%) versus 12.83% (SD: 6.24%); *p* = 0.023; Figs. 7 and 9). However, in S1 (16.83% (SD: 2.58%) vs. 14.59% (SD: 1.37%); *p* = 0.188) and S3 (18.66% (SD: 2.80%) vs. 16.90% (SD: 1.65%); *p* = 0.141), no significant differences were found between the groups.

**Fig. 10:**
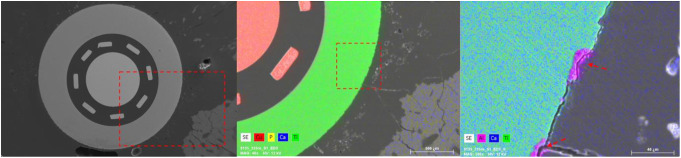
Illustrative scanning electron images are accompanied by EDX element analysis depicting titanium (green), copper (red), aluminum (pink), phosphate (yellow), and calcium (blue) distributions. The magnified sections are delineated by red rectangles, with arrows pinpointing regions where aluminum has been released from the titanium layer

Additional scanning electron images confirmed the integrity of the electrodes post-explantation. A detailed EDX element analysis revealed the preserved outer aluminum layer of the electrode, characterized by minimal instances of aluminum leakage (Fig. 10, pink spots indicated by arrows). In the lower right corner of Fig. 10, the emergence of new bone formation toward the electrode is evident, attributed to the heightened accumulation of calcium and phosphates.

## Discussion

The primary parameter of this study was the safety assessment of the novel ES implant device. No significant adverse effects were observed in the animals receiving the ES implants throughout the experimental period. Although two of the six animals experienced wound-healing complications three and four days after surgery, these issues occurred in an ES and a non-ES device. They were resolved through additional surgical exploration and re-fixation of the stimulation unit using the masseteric muscle. Following these interventions, no further complications were noted, and the animals continued to exhibit stable implant fixation with no signs of loosening or migration. These findings indicate that the novel ES implant system is safe for use in a large animal model, paving the way for future investigations into its efficacy in bone regeneration. While this study focused on the early-phase safety assessment of the electrically stimulated implant, a longer follow-up period would provide valuable insights into its long-term biocompatibility and bone regeneration potential. Given that the device can be designed for sustained function, future studies will extend the observation period to evaluate its effects on late-stage bone remodeling and implant integration. Future research will also explore resorbable or easily removable materials to enhance clinical applicability and reduce the need for secondary removal procedures.

In the present in vivo study, observations indicate that ES may possess the capacity to induce new bone formation in a critical-size defect. The promotion of osteogenesis did manifest substantial benefits of ES in overall terms when contrasted with the control group. The observed results can be attributed to the viable osseous cells near the ES implant, as bone particles were implanted in the defect. As elucidated earlier, ES primarily induces the osteogenic differentiation of mesenchymal stem cells (MSCs) [24, 25]. In the absence of these cells, as evident in a critical-size defect, the obtained outcomes appear logically explicable. The combination of tissue engineering and ES– i.e., using ES on tissue-engineered scaffolds with loaded MSCs - could be recommended for future research. In addition, a favorable and more prominent effect of ES was manifested within the original dense bone structure, as evidenced by a significantly higher total volume of bone in the stimulated group compared to the control group. This beneficial effect on osseointegration follows the literature [10, 36–39] but was demonstrated for the first time in an in vivo minipig model.

Regarding vascularization within the critical-size defect, a significantly higher number of vessels/erythrocytes was observed in the stimulated group compared to the control group. This finding aligns with the in vitro results of Kim et al., who demonstrated that biphasic ES enhances vascular endothelial growth factor (VEGF) production [40]. Additionally, ES has been shown to modulate the extracellular matrix (ECM) by upregulating collagen and fibronectin expression, both of which play essential roles in angiogenesis [41, 42]. ES also promotes angiogenesis in endothelial cells by increasing the expression of FRF2, HIF-1alpha, and VEGF [43–45]. Moreover, ES has been reported to directly induce pre-angiogenic responses in vascular endothelial cells through VEGF receptor activation, triggering downstream signaling cascades that promote vascular formation [46]. Studies further indicate that ES increases the secretion of pro-angiogenic factors such as VEGF and MCP-1, while simultaneously reducing anti-angiogenic factors like Serpin E1/PAI-1, shifting the balance toward enhanced vascularization [47]. Beyond its effects on gene expression, ES influences endothelial cell behavior via the PI3K-Akt and Rho-ROCK signaling pathways, which regulate cytoskeletal reorganization, cell migration, and alignment—all critical steps in angiogenesis [46]. Additionally, ES-induced angiogenesis may involve the angiotensin II receptor (AT1) pathway, suggesting a complex interplay of multiple regulatory mechanisms [48]. Furthermore, recent studies have highlighted that ES promotes angiogenesis via miRNA-mediated mechanisms, enhancing pro-angiogenic miRNA expression [38, 49, 50]. However, fibrous tissue typically exhibits increased vascularization, so a higher number of vessels alone may not necessarily indicate a positive outcome.

Upon scrutiny of mast cells in the proximity of ES and non-ES implants within the critical-size defect, an increase was evident in the stimulated samples, suggesting an active local tissue response. The immunological reaction following implant insertion plays a crucial role in determining the implant’s fate, and ES has been associated with the induction of M2 macrophage polarization and heightened macrophage activity, which are linked to pro-regenerative healing processes. This aligns with findings by Li et al., who reported that MSCs subjected to ES release diverse cytokines, fostering M2 macrophage polarization [49].

In the context of supplementary analysis involving soft tissue parameters, it is imperative to acknowledge that Toluidine blue staining is principally employed to visualize acidic tissue constituents, notably cartilage, and mast cells. While this staining technique provides valuable insights into certain aspects of soft tissue composition and inflammatory response, it does not specifically label bone tissue or blood vessels. Due to bone’s varying matrix composition (mineralization) in its developmental stages, the addition of Pyronine Yellow to Toluidine Blue results in differential staining. More mature bone binds less dye. Reducing the exposure time of the Toluidine Blue and Pyronine Yellow solution to the tissue makes distinguishing between different developmental or mineralization phases possible. However, interpretations of bone formation and vascularization results should be cautiously approached, as they rely primarily on histomorphometric assessments rather than specific markers for bone or blood vessels. Also, no clear identification of the inflammatory cells was conducted.

While the application of internal ES in human subjects remains scarce, the predominant body of in vivo research investigating the electrical stimulation of osseous tissues has been conducted utilizing rodent models [38, 51, 52]. Those exhibit notably accelerated bone healing rates compared to humans, rendering comparisons challenging [53]. The minipig has emerged as a viable animal model due to its physiological and anatomical similarities to humans, encompassing vascularization, fracture healing, and tissue regeneration [54, 55]. In brief, porcine bone resembles human bone and shows similar remodeling rates (pig, 1.2–1.5 mm per day; human, 1.0–1.5 mm per day) and lamellar bone microstructure [55, 56]. The application of ES for bone regeneration in a critical-size mandibular defect model in pigs has not been documented previously.

The three-week AC stimulation period was deliberate, aligning with the study’s objective of scrutinizing the implants’ safety. Prior in vitro analyses showed that long-term ES with 0.7 V and 20 Hz did lead to a beneficial response in human pre-osteoblasts without adverse effects [27]. In a canine model, Bins-Ely et al. substantiated that the osseointegration of ES implants exhibited a significant enhancement after 15 days of stimulation [10]. In rats, Huseynow et al. found an increase in reparative osteogenesis in electrically stimulated mandible defects after up to 28 days [38]. Moreover, Shayesteh et al. investigated the impact of ES on implant osseointegration within a comparable canine in vivo model, extending the evaluation period to 30 days. Their findings indicated a heightened bone contact ratio and local bone formation in the stimulated group compared to non-stimulated controls [36]. In an alternative canine model, a markedly increased maximum shear stress was documented in ES implants within the femur at 1, 2, and 3 weeks post-surgery [57]. In summary, the prevailing trend in other in vivo studies investigating ES for bone regeneration involves stimulation durations ranging from 2 to 6 weeks [52, 58].

While direct current (DC) stimulation– i.e., provided at a steady magnitude and direction– is the prevailing method for in vitro and in vivo applications targeting bone stimulation through ES [52, 59], numerous issues have been reported. For example, an unwanted accumulation of charged proteins at the electrode surface might obstruct current flow leading to inconsistent delivery of ES to the cells [19, 60], impairing bone tissue healing [61]. Moreover, DC stimulation might lead to the formation of reactive oxygen species that could even initiate bone resorption [62]. In contrast, the application of alternating current (AC) stimulation, characterized by a variable magnitude with periodic cycles, did not induce alterations in the characteristics of the cellular medium but was beneficial for the attachment and adhesion of osteoblasts in vitro [28]. Hence, the current study utilized AC signals, specifically sinusoidal stimulation, one of the most commonly employed forms of AC stimulation, alongside rectangular pulses [19].

Several in vitro studies have shown that continuous ES will lead to an initial increase in cellular proliferation but to a subsequent reduction of viable cells and their proliferation over a longer time [25, 63]. Dergin et al. conducted a study in an in vivo sheep model, employing continuous ES over 4, 8, and 12 weeks on titanium implants in the tibia. Their findings revealed no substantial differences between the test and control groups concerning the bone-to-implant ratio, osteoblast activity, and new bone formation [64]. Hence, as validated in vitro [23–25, 28], ES in the current investigation entailed a voltage-controlled sinusoidal application set at 0.5 V, featuring a frequency of 20 Hz. Stimulation was administered in 3 sessions lasting 45 min daily, with an intersession break of 225 min. These ES settings were successfully tested ex vivo and in vitro before [20, 23–26, 32].

While our study highlights the potential of ES to induce new bone formation within critical-size mandibular defects, it is essential to recognize the limitations of solely focusing on the observed acceleration of bone growth. Our results’ interpretations underscore ES’s positive effects on bone formation, vascularization, and immune response, as evidenced by histomorphometric assessments and previous literature. However, it is crucial to acknowledge that assessing additional parameters, such as bone mineralization, biomechanical properties, and long-term outcomes, would provide a more comprehensive understanding of the regenerative process. The promotion of osteogenesis observed in our study may be influenced by various factors beyond the scope of our investigation, including the absence of viable osseous cells and scaffolds near the ES implant and the dynamics of tissue regeneration in a critical-size defect environment. Moreover, while the observed acceleration of bone growth within the original dense bone structure is promising, validating these findings through complementary analyses is essential. In light of these considerations, future research endeavors should thoroughly evaluate multiple parameters to elucidate the full spectrum of ES-induced effects on bone regeneration. Incorporating techniques such as bone-specific labeling and biomechanical testing would enhance the rigor and validity of our conclusions, providing a more nuanced understanding of the efficacy of ES in promoting tissue healing and regeneration.

The limited sample size and inherent variability in biological organisms may cause a certain bias in this study, and it is essential to exercise caution in interpreting these results. Furthermore, the observed wound healing disorders, predominantly attributed to the positioning of the stimulation device and its fixation at the mandibular side, may have introduced bias into the results. Additionally, the titanium mesh employed in this study may not exhibit sufficient stability to prevent deformations, as observed in certain animals, potentially attributed to head movements. A further potential pitfall of electrical in vivo stimulation of osseous tissue is that the dielectric properties of the surrounding living tissue are not known unambiguously [65]. Besides, the body temperature might change, resulting in a changing electrical conductivity. Contrary to expectations, the hypothesis positing that incorporating an ES implant system into a critical-size mandibular defect would yield superior bone regeneration compared to a non-stimulated control group is deemed untenable based on empirical evidence. It is also important to acknowledge that our study’s lack of additional bone-specific labeling techniques, such as alizarin red or Von Kossa staining, represents a potential bias. While our pilot study primarily aimed to investigate the effects of direct ES on early bone regeneration within critical-size mandibular defects, the absence of these staining methods may limit the comprehensive assessment of bone formation and mineralization. Future research endeavors will incorporate these techniques to better understand the regenerative processes involved. Also, while this pilot study provides important insights into the safety and preliminary efficacy of electrically stimulated implants for mandibular bone regeneration, further research with larger sample sizes and extended follow-up periods is necessary to fully evaluate the clinical applicability of this approach.

## Conclusion

This study represents the first in vivo investigation demonstrating the safe application of electrical stimulation (ES) in a critical-size mandibular defect within a large animal model. ES had a significant impact on bone regeneration within the defect itself. In addition, it significantly enhanced vascularization and modulated the inflammatory response in the surrounding soft tissue. Also, when applied in pristine bone environments, ES promoted bone regeneration, highlighting its potential as an adjunctive tool in bone tissue engineering. Given the variability in study designs and ES parameters reported in the literature, further standardized in vivo research is essential to refine its clinical applicability. As this is an exploratory pilot study, long-term investigations with larger sample sizes are necessary before considering clinical translation.

## Electronic supplementary material

Below is the link to the electronic supplementary material.

Supplementary Material 1

## Data Availability

Data are available upon request from the corresponding author.
